# Effect of Curcumin on Diabetic Kidney Disease: A Systematic Review and Meta-Analysis of Randomized, Double-Blind, Placebo-Controlled Clinical Trials

**DOI:** 10.1155/2021/6109406

**Published:** 2021-12-02

**Authors:** Zhao Jie, Mo Chao, Ai Jun, Shi Wei, Meng LiFeng

**Affiliations:** ^1^Graduate School, Hunan University of Chinese Medicine, Changsha, Hunan 410208, China; ^2^Department of Nephrology, The First Affiliated Hospital of Guangxi University of Chinese Medicine, Nanning, Guangxi 530023, China; ^3^Graduate School, Guangxi University of Chinese Medicine, Nanning, Guangxi 530200, China; ^4^Basic Medicine School, Guangxi University of Chinese Medicine, Nanning, Guangxi 530200, China

## Abstract

**Background:**

Curcumin, a polyphenolic constituent from *Curcuma longa*, possesses antioxidant, hypolipidemic, and antidiabetic properties and has been reported to protect against diabetic kidney disease (DKD); however, the effect is inconsistent.

**Objective:**

This systematic review and meta-analysis aimed to investigate the effect of curcumin supplementation on renal function, lipid profile, blood pressure, and glycemic control in DKD.

**Methods:**

A systematic and comprehensive literature search of interrelated randomized controlled trials (RCTs) was conducted in PubMed, Embase, Cochrane Library, Web of Science, Scopus, and ClinicalTrials.gov from inception to July 30, 2021. Two investigators independently extracted data and assessed the risk of bias. Weighted mean differences (WMDs) with 95% confidence intervals (CIs) were calculated to describe the effect sizes using a fixed-effect model. Statistical analysis was performed using STATA 14.0 and RevMan 5.3.

**Results:**

Five RCTs involving 290 participants with DKD were included. Curcumin supplementation significantly improved the serum creatinine (WMD: −0.16 mg/dL, 95% CI: −0.3 to −0.02, *P* = 0.029, *I*^2^ = 0%, moderate certainty), total cholesterol (WMD: −10.13 mg/dL, 95% CI: −17.84 to −2.14, *P* = 0.01, *I*^2^ = 0%, moderate certainty), systolic blood pressure (WMD: 3.94 mmHg, 95% CI: 1.86 to 6.01, *P* < 0.01, *I*^2^ = 33.5%, moderate certainty), and fasting blood glucose (WMD: −8.29 mg/dL, 95% CI: −15.19 to −1.39, *P* = 0.019, *I*^2^ = 43.7%, moderate certainty) levels; however, it had no significant effects on blood urea nitrogen, proteinuria, triglyceride, low-density lipoprotein-cholesterol, high-density lipoprotein-cholesterol, and diastolic blood pressure levels.

**Conclusions:**

Curcumin may provide great potential effects against DKD. More large-scale and high-quality RCTs are required to confirm these findings.

## 1. Introduction

Diabetic kidney disease (DKD), a common microvascular complication of diabetes, is characterized by increased albuminuria level or urinary albumin-to-creatinine ratio (UACR), decreased glomerular filtration rate (GFR), or both [1]. According to the International Diabetes Federation data, approximately 463 million people were living with diabetes mellitus in 2019, and this number is projected to increase to 700 million by 2045 [2]. Up to 40% of patients with type 2 diabetes develop DKD, and DKD is currently the leading cause of end-stage renal disease (ESRD) worldwide [3]. Patients with DKD have a higher risk for cardiovascular morbidity and mortality, which are closely associated with risk factors such as hyperglycemia, dyslipidemia, and hypertension, than those without DKD [4]. DKD has become a considerable and growing challenge for health systems.

The introduction of lifestyle changes, for example, exercise and diet, optimal control of blood pressure (BP), glucose, and lipids, and treatment with pharmacological agents or pharmaceuticals, such as angiotensin-converting enzyme inhibitors and angiotensin receptor blockers, are recommended by clinical practice guidelines for the treatment of patients with DKD [5, 6]. Sodium-glucose cotransporter 2 inhibitors, a new class of antidiabetic agents, have now been shown to prevent major kidney outcomes in individuals with diabetes [3, 7]; however, close attention needs to be paid to their adverse effects [8]. Although these managements are effective, they do not seem to substantially decrease the morbidity of DKD and patients with this condition still have a high risk of disease progression [9], indicating the urgent need for complementary and alternative therapies.

Turmeric, a powder of the rhizomes of *Curcuma longa* L. (Zingiberaceae), is a well-known spice additive that is ubiquitous in Asian cuisine [10]. Turmeric is also a staple component of Ayurveda and traditional Chinese medicine and has been used as a remedy for various chronic diseases, such as cardiovascular disease and diabetes [11] and Alzheimer's disease [12]. Curcumin is a naturally occurring polyphenolic compound extracted from turmeric and has various pharmacological activities, including antioxidant, anti-inflammatory, immunomodulatory, hypolipidemic, glycemic control, and improving hepatic function as well as serum cortisol levels [13–15]. Glucose and lipid metabolism disorder, oxidative stress, and inflammation are among the major contributors to the pathogenesis of DKD [5], indicating that curcumin has properties that might improve the pathological changes in DKD. Animal experiments have revealed that curcumin administration in rats or mice with DKD could alleviate kidney damage by increasing antioxidant mediators [16], suppressing inflammatory factors [17], and decreasing proteinuria (PRO) [18]. Clinical trials have shown that curcumin supplementation is beneficial in improving renal function, lipid profile, BP, and fasting blood glucose (FBG) in patients with DKD [19–23]; however, contradictory results have been reported. Some studies reported that curcumin administration can significantly improve renal function [21, 22], lipid profile [19], BP [19], and FBG levels [19, 20], whereas other trials reported contrasting results [20, 23]. Therefore, this study aimed to perform a comprehensive review and meta-analysis of previous pilot, double blind, placebo-controlled, randomized controlled trials (RCTs) on the effect of curcumin supplementation on DKD.

## 2. Materials and Methods

This meta-analysis was performed according to the guidelines of Preferred Reporting Items for Systematic Review and Meta-Analysis (PRISMA) [24] and was registered at INPLASY (https://inplasy.com/) with a registration ID INPLASY202180001. The checklist of PRISMA is shown in Supplementary Table S1.

### 2.1. Search Strategy

The literature was systematically searched using electronic databases, including PubMed, Embase, Cochrane Library, and Web of Science and a clinical trial registry (ClinicalTrials.gov), from inception to July 30, 2021. Medical Subject Heading terms and text words were used as search terms, including “curcumin,” “turmeric,” “diabetic,” “diabetic nephropathies,” “diabetic kidney disease,” and “randomized controlled trial.” All searches were limited to English-language publications. We also manually screened the reference list of eligible studies in case of missing appropriate studies. The detailed search strategies for databases are presented in Supplementary Table S2.

### 2.2. Study Selection

A trial was included if it met the following criteria: (i) designed as an RCT; (ii) included patients diagnosed with DKD, defined as urinary albumin-to-creatinine ratio ≥30 mg/g or albumin excretion rate ≥30 mg/24 h or estimated GFR (eGFR) <60 mL/min/1.73 m^2^ for ≥3 months caused by diabetes mellitus [25] (no restrictions on the patients' sex, region, race, and disease course were implemented); (iii) administered curcumin to participants in the intervention group and placebo to the control group (with no limitation in dosage or follow-up period); and (iv) reported at least one of the following outcomes: renal function parameters: including serum creatinine (SCr), blood urea nitrogen (BUN), and PRO; lipid profile, including total cholesterol (TC), triglycerides (TG), low-density lipoprotein-cholesterol (LDL-C), high-density lipoprotein-cholesterol (HDL-C); BP, including systolic blood pressure (SBP) and diastolic blood pressure (DBP); and FBG levels. A study was excluded if it met the following criteria: (i) included participants not diagnosed with DKD; (ii) performed the trial in animals; (iii) published as an observational study, case report, meta-analysis, or review; and (iv) included participants who received drugs or supplements other than curcumin.

### 2.3. Data Extraction

Two investigators (J.Z. and C.M.) independently screened eligible studies according to predetermined criteria. One investigator (J.Z.) extracted the following data from each study: study characteristics, including first author, publication year, country, and study design; participant characteristics, including sample size, mean age, and sex ratio; interventions and comparison, including the use and dosage of turmeric/curcumin and the follow-up period; and available outcomes. The other investigator (C.M.) rechecked the data for consistency. Any discrepancy between the two investigators (J.Z. and C.M.) was resolved through a discussion with a third investigator (J.A.). For missing data, we tried our best to contact corresponding authors via e-mail.

### 2.4. Risk of Bias Assessment

Two investigators (J.Z. and C.M.) independently assessed the risk of bias of the included studies using the Cochrane Risk of Bias tool [26], including six items: selection bias, performance bias, detection bias, attrition bias, reporting bias, and bias from other sources. These items were judged as “low risk,” “high risk,” or “unclear.” Any disagreements were resolved through a discussion with a third investigator (J.A.).

### 2.5. Data Synthesis and Analysis

The effects of outcomes in the intervention and placebo groups were estimated by comparing the changes in mean differences before and after therapy (i.e., end values minus baseline values). The standard deviation (SD) changes in outcomes were calculated according to the following formula: SD = sqrt [(SD before treatment) 2 + (SD after treatment) 2–(2R × SD before treatment × SD after treatment)], where *R*, a correlation coefficient, was equal to 0.5 [27]. Weighted mean differences (WMDs) with corresponding 95% confidence intervals (CIs) for continuous outcomes were calculated to describe the effect sizes. Cochran's *Q* and I2 statistics were employed to evaluate statistical heterogeneity. *I*^2^ values of <25%, >50%, and >75% were considered to indicate low, moderate, and high heterogeneity, respectively [28]. Participants (≤60 versus > 60 years), dosage of curcumin supplementation (≥1500 versus < 1500 mg/day), and follow-up period (≤2 versus > 2 months) or sensitivity analysis was conducted to identify the sources of heterogeneity. Furthermore, sensitivity analysis was performed to evaluate the robustness of outcomes. For outcomes with small heterogeneity, sensitivity analyses were performed using the conversion effect model. In contrast, if outcomes had considerable heterogeneity, sensitivity analysis was performed by omitting one study at a time. Begg's and Egger's tests were used to examine potential publication bias [29]. Statistical analysis was performed using STATA 14.0 (StataCorp, College Station, TX, USA) and RevMan 5.3. *P* values <0.05 were considered statistically significant.

### 2.6. GRADE Assessment

Two investigators (J.Z. and C.M., W.S., or J.A.) independently evaluated the certainty of the evidence for outcomes according to the GRADE system, an approach for rating the quality of evidence and strength of recommendations, graded as high, moderate, low, or very low certainty [30]. GRADE assessments were downgraded according to the following criteria: (i) risk of bias was downgraded if one of the items used to evaluate the risk of bias was considered to be at a high risk of bias and most of the included studies were identified as having a high risk of bias; (ii) imprecision was downgraded if the 95% CIs for effect estimates overlap zero for continuous outcomes; (iii) inconsistency was downgraded if there was substantial heterogeneity (*I*^2^ > 50% and *P* < 0.1) that was unexplained by any subgroup or sensitivity analysis; (iv) indirectness was downgraded if there were any influencing factors that limited the interpretation of outcomes; and (v) publication bias was downgraded if there was a significant difference in the evidence of publication bias according to either Begg's test or Egger's test (*P* < 0.05).

## 3. Results

### 3.1. Description of Included Studies

**Figure 1:**
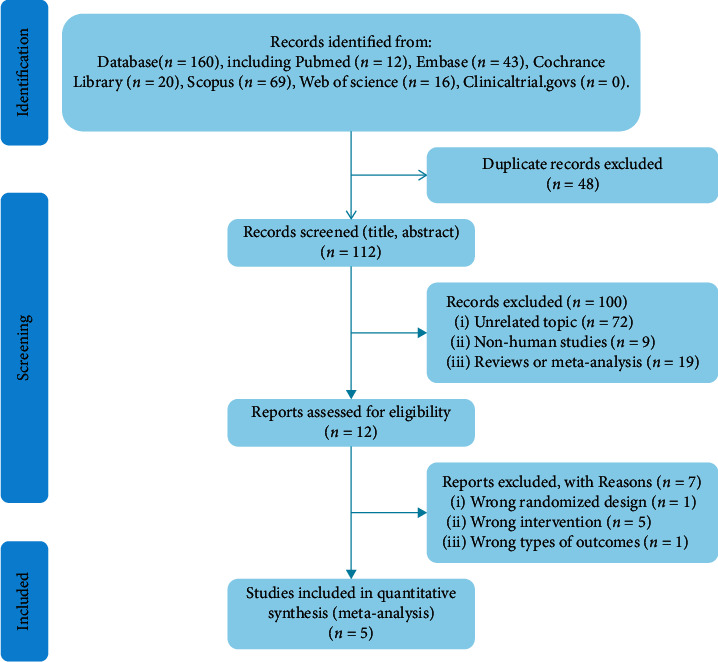
The PRISMA flow diagram.

A total of 160 potentially relevant records were obtained from PubMed (*n* = 12), Embase (*n* = 43), Cochrane Library (*n* = 20), Scopus (*n* = 69), Web of Science (*n* = 16), and ClinicalTrials.gov (*n* = 0). After removing 48 duplicates, 112 records remained for screening. According to predefined inclusion criteria, 100 records were excluded after screening the titles and abstracts. Consequently, after full-text assessment and detailed examination, seven records were excluded because of having an inappropriate study design, using an inappropriate intervention, or not reporting the data of interest. Ultimately, five RCTs [19–23] were included. The PRISMA flow diagram is shown in Figure 1.

### 3.2. Characteristics of Included Studies

Overall, five trials involving 290 patients were included. These trials were conducted in Iran and Mexico and were published between 2011 and 2020. Two trials included patients with overt diabetic nephropathy [21, 22]; two trials were performed in patients with diabetic proteinuric chronic kidney disease [20, 23]; and one trial enrolled patients with diabetes requiring hemodialysis [19]. All included studies were randomized, double-blind, placebo-controlled trials. The sample size varied from 20 to 54. The mean age of patients was 60 years. Three trials used curcumin capsules at doses ranging from 320 to 1670 mg/day [20, 22, 23]. One study used turmeric capsules that included curcumin at a dose of 66.3 mg/day [21], whereas another trial used nanocurcumin capsules at a dose of 80 mg/day [19]. The follow-up periods ranged from 2 to 6 months. The main characteristics of included trials are summarized in Table 1.

### 3.3. Methodological Quality Assessment

**Figure 2:**
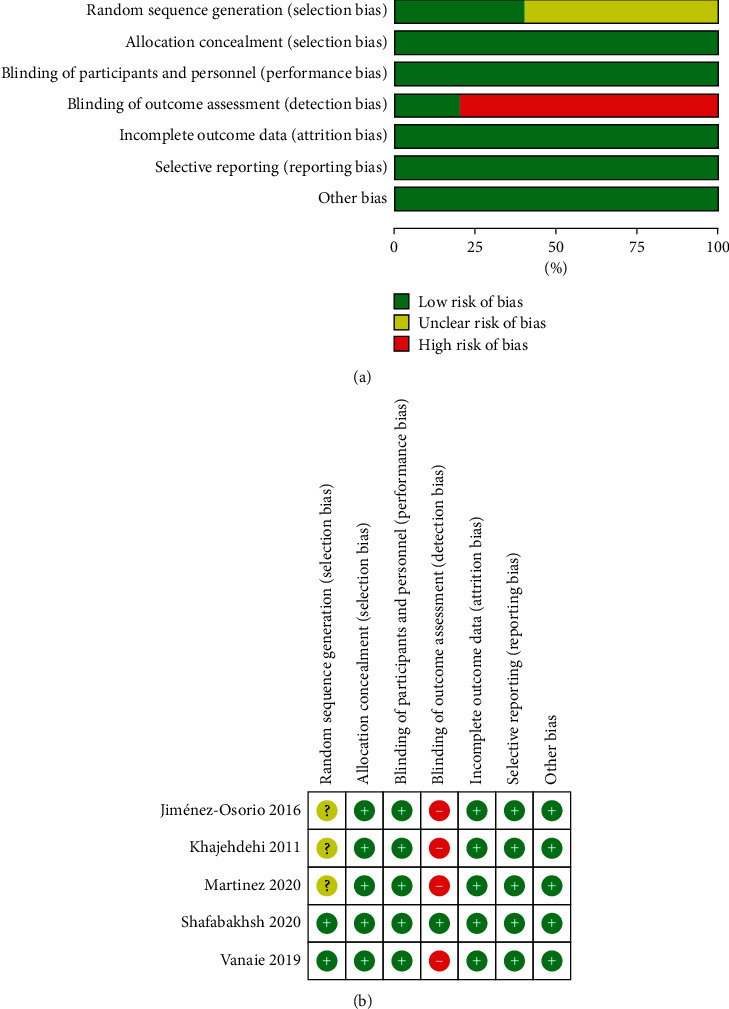
Risk of bias of included trials. (a) represents the risk of bias graph, and (b) represents the risk of bias summary.

All studies had a randomized parallel design. Two trials (40%) showed a low risk of bias and used permuted blocks [22] and computer-generated random numbers [19], respectively. The remaining three RCTs were classified as having an unclear risk of bias owing to inadequate descriptions of randomization methods [20, 21, 23]. With respect to allocation concealment, blinding of participants and researchers, incomplete outcome data, selective reporting, and other biases, all included trials were classified as having a low risk of bias. Notably, four trials (80%) that did not blind outcomes were assessed as having a high risk of bias [20–23]. The risk of bias is presented in Figure 2.

### 3.4. Meta-Analysis of Outcomes

#### 3.4.1. Effects of Curcumin Supplementation on Renal Function

**Figure 3:**
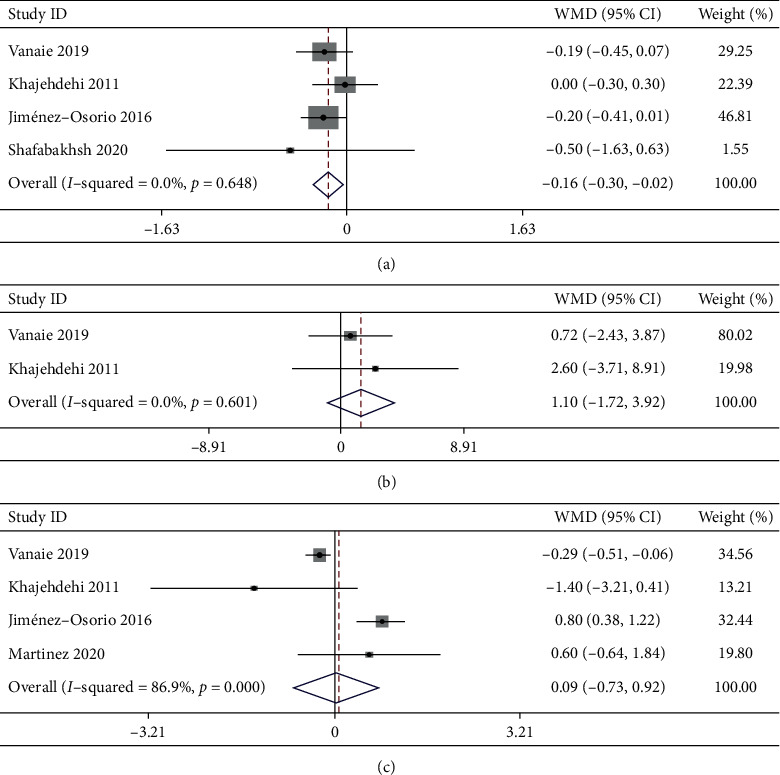
Effects of curcumin supplementation on renal function. (a) Serum creatinine; (b) blood urea nitrogen; (c) proteinuria.

3.4.1.1 *SCr*. Four trials with 190 participants reported the effects of curcumin supplementation on SCr. Compared with placebo, curcumin supplementation was associated with a significant decrease in SCr levels (WMD: −0.16 mg/dL, 95% CI: −0.3 to −0.02, *P* = 0.029; *I*^2^ = 0%, *P*_heterogeneity_ = 0.648; Figure 3(a) and Table 2).

3.4.1.2 *BUN*. Three trials with 139 participants studied the effect of curcumin on BUN. The pooled effect of curcumin administration showed no significant difference in BUN (WMD: −0.80 mg/dL, 95% CI: −5.62 to 4.02, *P* = 0.933), with high heterogeneity (*I*^2^ = 60% and *P*_heterogeneity_ = 0.082). Subgroup analysis showed that the effect of curcumin supplementation on BUN also had substantial heterogeneity in all the analyzed subgroups (Table 3). Further sensitivity analysis revealed that the heterogeneity of *I*^2^ = 60% changed to *I*^2^ = 0% after removing the study by Shafabakhsh et al. [19], which was probably the major source of the heterogeneity. When the other two trials with 86 participants were pooled, the revised result showed that the effect of curcumin on BUN still had no significant difference from that of placebo (WMD: 1.10 mg/dL, 95% CI: −1.72 to 3.92, *P* = 0.446; *I*^2^ = 0%, *P*_heterogeneity_ = 0.601; Figure 3(b) and Table 2).

3.4.1.3 *PRO*. Four trials with 237 participants assessed the effect of curcumin on PRO. No significant difference in PRO was observed with curcumin supplementation (WMD:0.09 g/24 h, 95% CI: −0.73 to 0.92, *P* = 0.614), with considerable heterogeneity between studies (*I*^2^ = 86.9%, *P*_heterogeneity_＜0.01) (Figure 3(c) and Table 2). Subgroup analysis revealed that the statistical heterogeneity decreased in participants with age ≤60 years (*I*^2^ = 41.4% and *P*_heterogeneity_ = 0.181), dosage of curcumin intake ≥1500 mg/day (*I*^2^ = 47.4% and *P*_heterogeneity_ = 0.168), and follow-up period >2 months (*I*^2^ = 47.4% and *P*_heterogeneity_ = 0.168), suggesting that these factors may be the sources of the heterogeneity (Table 3).

#### 3.4.2. Effects of Curcumin Supplementation on Lipid Profile

**Figure 4:**
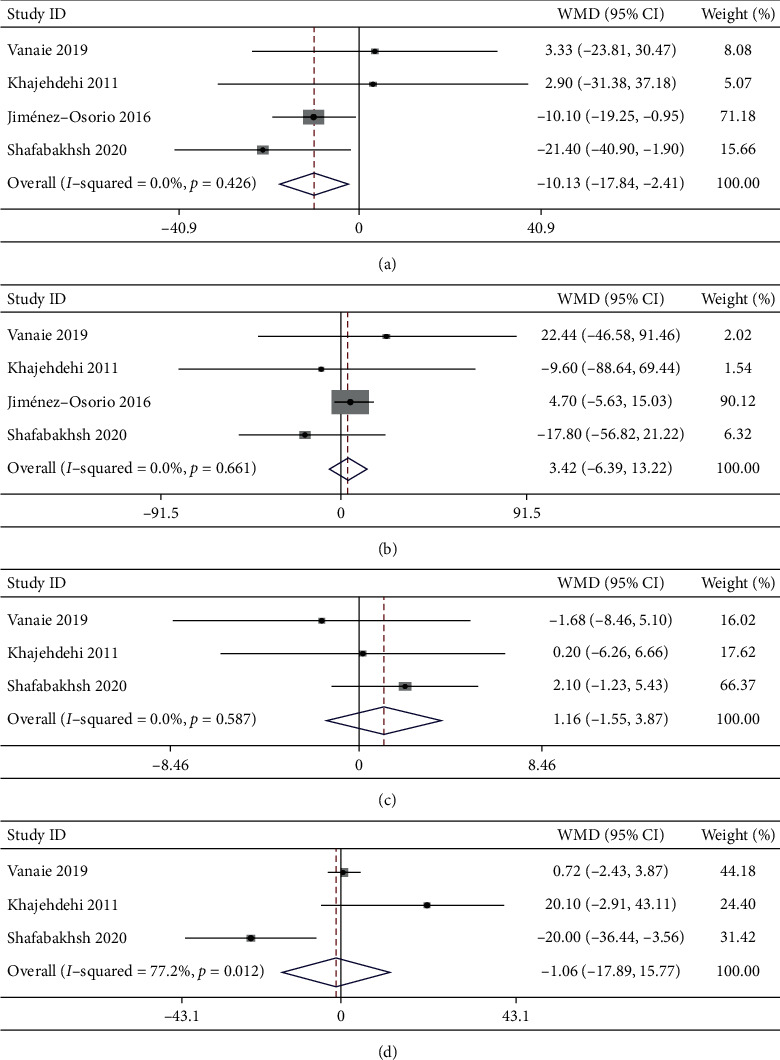
Effects of curcumin supplementation on lipid profile. (a) Total cholesterol; (b) triglycerides; (c) high-density lipoprotein-cholesterol; (d) low-density lipoprotein-cholesterol.

3.4.2.1 *TC*. Four trials with 190 participants analyzed the impact of curcumin supplementation on TC. The result of the meta-analysis showed a significant decrease in TC after curcumin administration (WMD: −10.13 mg/dL, 95% CI: −17.84 to −2.14, *P* = 0.01; *I*^2^ = 0%, *P*_heterogeneity_ = 0.426; Figure 4(a) and Table 2).

3.4.2.2 *TG*. Four trials with 190 participants reported the effect of supplemental curcumin on TG. No significant difference in TG was observed after curcumin administration (WMD: 3.42 g/dL, 95% CI: −6.93 to 13.22, *P* = 0.495; *I*^2^ = 0%; *P*_heterogeneity_ = 0.661; Figure 4(b) and Table 2).

3.4.2.3 *HDL-C*. Three trials with 139 participants assessed the effects of curcumin on HDL-C. Compared with placebo, curcumin supplementation showed a nonsignificant effect on HDL-C (WMD: 1.16 mg/dL, 95% CI: −1.55 to 3.87, *P* = 0.402; *I*^2^ = 0%, *P*_heterogeneity_ = 0.587; Figure 4(c) and Table 2).

3.4.2.4 *LDL-C*. Three trials with 139 participants assessed the effects of curcumin on LDL-C. The meta-analysis showed that curcumin supplementation did not affect changes in LDL-C (WMD: −1.06 mg/dL, 95% CI: −17.89 to 15.77, *P* = 0.902; *I*^2^ = 77.2%, *P*_heterogeneity_ = 0.012; Figure 4(d) and Table 2). The considerable heterogeneity between studies remained after subgroup analysis according to the mean age of participants, dosage of curcumin, and follow-up period (Table 3). The sensitivity analysis also did not explain the heterogeneity. After carefully reading the original articles, we found that the participants in the three studies were different, although they were all diagnosed with DKD. Participants with overt type 2 diabetic nephropathy were included in two studies, and patients with diabetes undergoing hemodialysis were enrolled in the other trial, and this difference was perhaps the source of the heterogeneity.

#### 3.4.3. Effects of Curcumin Supplementation on BP

**Figure 5:**
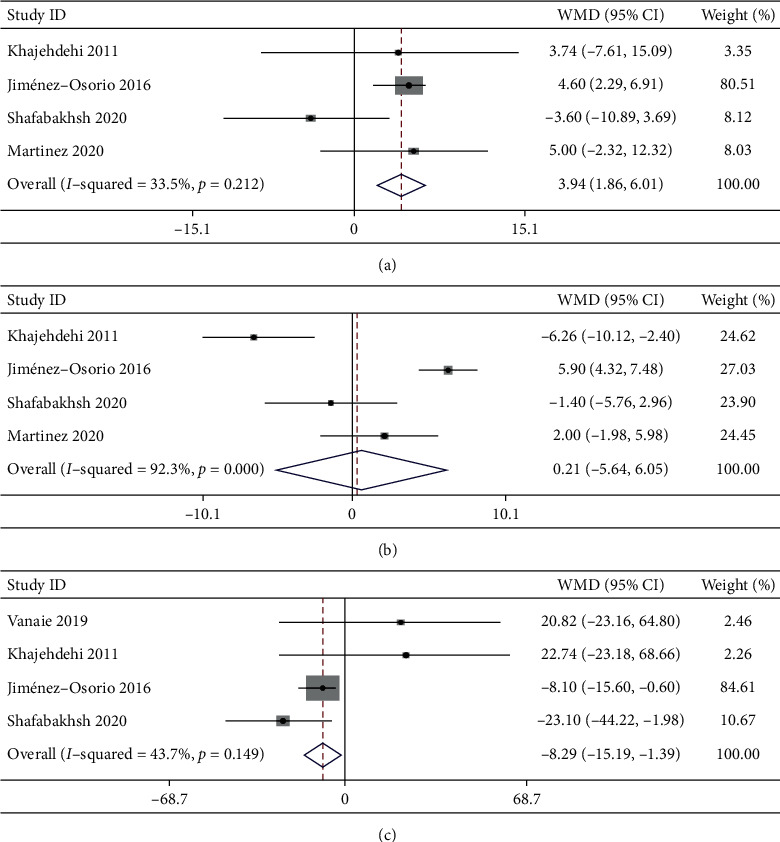
Effects of curcumin supplementation on blood pressure and glycemic control. (a) Systolic blood pressure; (b) diastolic blood pressure; (c) fasting blood glucose.

3.4.3.1 *SBP*. Four trials with 244 participants investigated the effect of curcumin supplementation on SBP. The pooled effect size of curcumin supplementation for SBP was 3.94 mmHg (95% CI: 1.86 to 6.01, *P* = 0.00), with heterogeneity between studies (*I*^2^ = 33.5%, *P*_heterogeneity_ = 0.212) for SBP (Figure 5(a) and Table 2).

3.4.3.2 *DBP*. Four trials with 244 participants investigated the effect of curcumin supplementation on DBP. The differences in DBP were not statistically significant (WMD: 0.21 mmHg, 95% CI: −5.64 to 6.05, *P* = 0.944; *I*^2^ = 92.3%, *P*_heterogeneity_＜0.001) between the curcumin group and the placebo group (Figure 5(b) and Table 2). Heterogeneity between studies was insignificant (*I*^2^ = 21.4%, *P*_heterogeneity_ = 0.259) after subgroup analysis according to follow-up period (>2 months), which was probably the source of the heterogeneity (Table 3).

#### 3.4.4. Effects of Curcumin Supplementation on FBG

Four trials with 190 participants analyzed the impact of curcumin supplementation on FBG. The overall effect size revealed a significant decrease in FBG after curcumin administration (WMD: −8.29 mg/dL, 95% CI: −15.19 to −1.39, *P* = 0.019; *I*^2^ = 43.7%, *P*_heterogeneity_ = 0.149; Figure 5(c) and Table 2).

### 3.5. Sensitivity Analysis

For outcomes with insignificant heterogeneity (SCr, TC, TG, and HDL-C), no significant impact of the conversion effect model on the overall effect sizes was observed. With respect to outcomes with substantial heterogeneity (BUN, PRO, LDL-C, and DBP), the overall effect sizes were not significantly affected when any single study was omitted. These indicated the robustness of the combined result. However, the overall effect sizes for outcomes with insignificant heterogeneity (SBP and FBG) were influenced by the conversion effect model, indicating the poor robustness of SBP and FBG as outcomes. Therefore, the results on SBP and FBG should be interpreted with caution.

### 3.6. Publication Bias

No evidence of publication bias was observed for the effects of curcumin supplementation on SCr, BUN, PRO, TC, TG, HDL-C, LDL-C, SBP, DBP, and FBG based on Begg's test and Egger's test (Supplementary Table 2S3).

### 3.7. GRADE Assessment

The overall certainty of the evidence for the effects of curcumin supplementation on renal function, lipid profile, BP, and glycemic control is presented in Table 4. The quality of the evidence for SCr, TC, SBP, and FBG was graded as “moderate” after being downgraded for risk of bias. Meanwhile, the quality of the evidence for BUN, PRO, TG, HDL-C, and DBP was graded as “low” after being downgraded for risk of bias and imprecision. The quality of the evidence for LDL-C was downgraded for risk of bias, inconsistency, and imprecision and was graded as “very low.”

## 4. Discussion

To our knowledge, this is the first meta-analysis of RCTs to analyze the effects of curcumin supplementation on DKD. Our findings showed that supplemental curcumin, in comparison with placebo, significantly improved the SCr, TC, SBP, and FBG levels in patients with DKD, with moderate certainty of evidence; however, it had no significant effects on the BUN, PRO, TG, HDL-C, LDL-C, and DBP levels.

The presence of PRO indicates renal parenchymal injury, which has been considered a clinical hallmark and a sign of renal dysfunction [31]. As PRO increases, eGFR decreases, resulting in end-stage renal disease and an increased risk of cardiovascular events [32, 33]. It is generally known that elevated levels of SCr and BUN in serum also indicate renal impairment. In the current meta-analysis, curcumin supplementation significantly decreased the SCr levels. Nevertheless, no effects of curcumin on BUN and PRO were observed in patients with DKD, consistent with the findings of a previous systematic review that investigated the role of curcumin in the treatment of renal disorders [34]. However, evidence from animal studies showed that curcumin significantly reduced the levels of SCr, BUN, and PRO [18], indicating that the differences in the effects of curcumin between animal experiments and clinical trials need further verification.

Lipid accumulation in podocytes results in podocyte injury and renal impairment and has been considered a driving factor for the development and progression of DKD [35]. Dyslipidemia is positively related to atherosclerosis and increases the risk of cardiovascular disease [36], indicating the urgent need for the management of dyslipidemia. Curcumin has been widely investigated owing to its obvious hypolipidemic effects. A study showed that curcumin significantly reduced the elevated levels of TC and TG and increased the level of HDL-C in rats fed a high-fat diet by increasing hepatic fatty acid oxidation activity and improving the apoptotic status of liver tissue [37]. In addition, the hypolipidemic effects of curcumin are also attributed to the suppression of the rate-limiting enzyme in the TC synthesis pathway; upregulation of the expressions of ABCA1, Apo-A1, and SR-BI, which are associated with reverse TC transport through HDL-C particles; induction of LDL-C-receptor expression; and inhibition of ApoB100 expression [38]. Meta-analyses of RCTs reported that curcumin supplementation showed benefits on lipid metabolism in patients with type 2 diabetes [39], polycystic ovary syndrome [14], and nonalcoholic fatty liver disease [40]. Our findings demonstrated a remarkable reduction in the TC level after curcumin administration in patients with DKD. However, the results should be interpreted with caution owing to the limited number of studies, and larger-scale trials are required to verify the results.

Hypertension is also closely associated with CKD development and progression [1]. It has been demonstrated that a 10–20 mmHg increase in SBP increases the risk of DKD by 21% [41]. Therefore, reducing BP, particularly SBP, can help decrease the risk of DKD. The effect of curcumin supplementation on BP is not fully conclusive. Damoon et al. reported that the hypotensive effect of nanocurcumin on cardiovascular disease is mainly focused on decreasing SBP [42]. Amir et al. observed favourable effects of curcumin administration only on SBP levels in different participants with a ≥12-week follow-up [43]. In contrast, Maryam et al. observed that curcumin supplementation improved DBP in participants with metabolic syndrome; however, it was not associated with a change in SBP [44]. The result of the present meta-analysis indicated that curcumin administration had a prominent effect on SBP in patients with DKD. We speculate that the discrepancy in the effects of curcumin supplementation on BP may be associated with differences in participants and follow-up periods across studies. A few potential mechanisms have been proposed to explain the beneficial effects of curcumin on BP. Curcumin prevents the development of hypertension by downregulating angiotensin II type-1 receptor (AT1R) expression to alleviate AT1R-mediated vasoconstriction [45]. In addition, curcumin inhibits vascular smooth muscle cell (VSMC) migration by inhibiting NF-*κ*B-mediated NLRP3 expression in angiotensin II-treated VSMCs [46].

Hyperglycemia is also identified as an important factor for CKD development beyond its links to lipid accumulation and hypertension [1]. Intensive glycemic therapy, such as controlling the haemoglobin A1c levels within 6.5–7.0%, contributes to reducing the risk of DKD [47]. Evidence from both in vivo and vitro studies indicated a strong potential effect of curcumin against insulin resistance and diabetes [48, 49]. Previous meta-analyses showed that curcumin significantly improved FBG levels in individuals with polycystic ovary syndrome [14], some degree of dysglycemia [50], and metabolic syndrome [44]. Consistent with these findings, we also observed conspicuous effects of curcumin supplementation on FBG levels in patients with DKD, adding to the existing evidence on the benefits of curcumin in improving FBG. However, there is no evidence about the impact of curcumin supplementation on other glycemic parameters, such as haemoglobin A1c, serum insulin, and homeostasis model assessment-insulin resistance index in the included trials. Large-scale studies focusing on these topics are needed in the future.

### 4.1. Strengths and Limitations

To our knowledge, this is the first meta-analysis of RCTs to assess the effects of curcumin supplementation on DKD and to provide evidence for curcumin as a promising agent in the treatment of this condition. We conducted the current study strictly according to the PRISMA guidelines and observed significant effects of curcumin on SCr, TC, SBP, and FBG levels. Furthermore, we evaluated the certainty of the evidence for outcomes using the GRADE method. The evidence for SCr, TC, SBP, and FBG was graded as having moderate certainty.

Several limitations should be considered when interpreting our findings. First, the sample sizes and the number of trials that were eligible for inclusion were small. Second, various dosages and durations of treatment were applied for the intervention. Third, the adverse effects of curcumin require attention; however, it is difficult to assess the safety of curcumin owing to the limited number of reports on its potential adverse effects. Finally, three (60%) trials did not provide adequate information on randomization methods and four (80%) trials did not perform blinding of outcomes, which may have reduced the quality of the evidence of the studies.

### 4.2. Implications for Practice

According to the available evidence from this meta-analysis, curcumin supplementation has beneficial effects on SCr, TC, SBP, and FBG levels, with moderate certainty, in patients with DKD and seems to be a promising agent against DKD. Nevertheless, our results should be interpreted with caution considering the above-mentioned limitations.

### 4.3. Implications for Research

As the results of meta-analyses are closely related to evidence-based treatment decisions in the clinic, rigorously designed studies are highly warranted. Large-sample, multicentre, high-quality, and well-designed clinical trials should be registered to ensure the transparency of the process and improve the methodological quality. Moreover, future studies should also focus on adverse effects, apart from efficacy, to evaluate the safety of curcumin.

## 5. Conclusions

In conclusion, the current study shows that curcumin supplementation provides evident improvements in the SCr, TC, DBP, and FBG levels in patients with DKD, with moderate certainty of evidence. However, although curcumin supplementation has great potential effects on DBP and FBG, the results should be interpreted with caution because of the poor robustness of the evidence. The effect of curcumin supplementation on DKD should be confirmed by more large-scale and high-quality RCTs.

## Figures and Tables

**Table 1:** Summary of characteristics of included studies.

First author, publication year, country	Study characteristics		Population characteristics				Intervention and compairion		Follow-up time (M)	Outcomes			
	Study design	Participants	Sample sizes (I/C)	Mean age (years, I/C)	Males (%, I/C)	Mean duration of DM (years, I/C)	Intervention group	Control group		Renal function	Lipid profile	Glycemic control	Blood pressure
Khajehdehi 2011, Iran	Randomized, double-blind, placebo-controlled clinical trial	DKD	20/20	52.9/52.6	45/65	NA	Turmeric capsule 1500 mg/day, which included curcumin 66.3 mg/day	Placebo	2	SCr, PRO, BUN	TC, TG, HDL-C, LDL-C	FBG	SBP, DBP
Jiménez-Osorio 2016, Mexico	Randomized, double-blind, placebo-controlled clinical trial	DKD	28/23	73.9/67.9	55/56.2	NA	Curcumin capsule 320 mg/day	Placebo	2	SCr, PRO	TC, TG	FBG	SBP, DBP
Vanaie 2019, Iran	Randomized, double-blind, placebo-controlled clinical trial	DKD	27/19	59/61	59/58	16/15	Curcumin capsule 1500 mg/day	Placebo	4	SCr, BUN, PRO	TC, TG, HDL-C, LDL-C	FBG	NA
Shafabakhsh 2020, Iran	Randomized, double-blind, placebo-controlled clinical trial	DKD	26/27	58.3/56.2	65.4/55.6	NA	Nanocurcumin capsule 80 mg/d	Placebo	3	SCr, BUN	TC, TG, HDL-C, LDL-C	FBG	SBP, DBP
Martinez 2020, Mexico	Randomized, double-blind, placebo-controlled clinical trial	DKD	54/46	59.3/57.4	57.4/78.3	NA	Curcumin 1670 mg/day	Placebo	6	PRO	NA	NA	SBP, DBP

SCr, serum creatinine; BUN, blood urea nitrogen; PRO, proteinuria; TC, total cholesterol; TG, triglycerides; LDL-C, low-density lipoprotein-cholesterol; HDL-C, high-density lipoprotein-cholesterol; SBP; systolic blood pressure; DBP, diastolic blood pressure; DKD, diabetic kidney disease; FBG, fasting blood glucose; NA, not available; I, intervention group; C, control group; *M*, month.

**Table 2:** Effect of curcumin supplementation on outcomes.

Outcomes	No. of studies	Sample size	Heterogeneity		Analysis model	WMD (95% CI)	*Pvalue*
			*I* ^2^	*P heterogeneity*			
**Renal function**							
SCr	4	190	0%	0.648	Fixed	−0.16 (−0.3 to −0.02)	**0.029**
BUN	2	86	0%	0.601	Fixed	1.10 (−1.72 to 3.92)	0.446
PRO	4	237	86.90%	＜0.01	Random	0.09 (−0.73 to 0.92)	0.614

**Lipid profile**							
TC	4	190	0%	0.426	Fixed	−10.13 (−17.84 to −2.14)	**0.01**
TG	4	190	0%	0.661	Fixed	3.42 (−6.93 to 13.22)	0.495
HDL-C	3	139	0%	0.587	Fixed	1.16 (−1.55 to 3.87)	0.402
LDL-C	3	139	77.20%	0.012	Random	−1.06 (−17.89 to 15.77)	0.902

**Blood pressure**							
SBP	4	244	33.50%	0.212	Fixed	3.94 (1.86 to 6.01)	＜0.01
DBP	4	244	92.30%	＜0.001	Random	0.21 (−5.64 to 6.05)	0.944

**Glycemic control**							
FBG	4	190	43.70%	0.149	Fixed	−8.29 (−15.19 to −1.39)	**0.019**

SCr, serum creatinine; BUN, blood urea nitrogen; PRO, proteinuria; TC, total cholesterol; TG, triglycerides; LDL-C, low-density lipoprotein-cholesterol; HDL-C, high-density lipoprotein-cholesterol; SBP, systolic blood pressure; DBP, diastolic blood pressure; FBG, fasting blood glucose.

**Table 3:** Subgroup analysis to assess the effect of curcumin supplementation on blood urea nitrogen, proteinuria, low-density lipoprotein-cholesterol, and diastolic blood pressure.

Outcomes		Overall effects	Subgroups analyzed					
			Mean age		Curcumin intake		Follow-up period	
			≤60 years	>60 years	≥1500 mg/day	＜1500 mg/day	≤2 months	＞2 months
**Blood urea nitrogen**	No. of trials	3	3	—	1	2	1	2
	WMD (95% CI)	−0.8 (−5.62, 4.02)	−0.11 (−2.71, 2.49)		0.72 (−2.43, 3.87)	−1.12 (−1.53, 7.28)	2.60 (−3.71, 8.91)	−2.54 (−10.02, 4.93)
	*Pvalue*	0.933	0.933		0.654	0.658	0.419	0.505
	*I* ^2^ (%)	60	60		—	75.9	—	75.9
	*Pheterogeneity*	0.082	0.082		—	0.041	—	0.042

**Proteinuria**	No. of trials	4	3	1	2	2	2	2
	WMD (95% CI)	−0.09 (−0.73, 0.92)	−0.23 (−0.95, 0.05)	0.80 (0.38, 1.22)	−0.06 (−0.82, 0.69)	−0.12 (−2.24, 2.01)	−0.12 (−2.24, 2.01)	−0.06 (−0.82, 0.69)
	*Pvalue*	0.823	0.536	＜0.001	0.875	0.914	0.914	0.875
	*I* ^2^ (%)	86.90%	41.40%	—	47.40%	81.50%	81.50%	47.40%
	*Pheterogeneity*	＜0.001	0.181	—	0.168	0.002	0.02	0.168

**Low-density lipoprotein-cholesterol**	No. of trials	3	3	—	1	2	1	2
	WMD (95% CI)	−1.06 (−17.89, 15.77)	0.34 (−2.73, 3.41)		0.72 (−2.43, 3.87)	−0.79 (−0.05, 38.47)	20.10 (−2.91, 43.11)	−8 (−28.05, 12.05)
	*Pvalue*	0.902	0.902		0.654	0.968	0.087	0.434
	*I* ^2^ (%)	77.2	77.2		—	87	—	83
	*Pheterogeneity*	0.012	0.012		—	0.005	—	0.015

**Diastolic blood pressure**	No. of trials	4	3	1	1	3	2	2
	WMD (95% CI)	0.21 (−5.64, 6.05)	−1.91 (−6.79, 2.96)	5.90 (4.32, 7.48)	2.00 (−1.98, 5.98)	−0.44 (−8.51, 7.63)	−0.05 (−11.96, 11.87)	0.42 (−2.90, 3.75)
	*Pvalue*	0.944	0.441	＜0.001	0.325	0.915	0.803	0.994
	*I* ^2^ (%)	92.3	76.8	—	—	94.8	96.9	21.4
	*Pheterogeneity*	＜0.001	0.013	—	—	＜0.001	＜0.001	0.259

WMD, weight mean difference; CI, confidence interval.

**Table 4:** The certainty of the evidence for the effects of curcumin supplementation on outcomes based on the GRADE assessment.

Outcomes	No. of trials	Study design	Quality assessment					Effect of WMD (95% CIs)	Quality
			Risk of bias	Inconsistency	Indirectness	Imprecision	Publication bias		
**Renal function**									
SCr	4	RCTs	Serious^a^	Not serious	Not serious	Not serious	Not serious	−0.16 (−0.30, −0.02)	⨁⨁⨁◯ moderate
BUN	2	RCTs	Serious^a^	Not serious^b^	Not serious	Serious^c^	Not serious	1.10 (−1.72, 3.92)	⨁⨁◯◯ low
PRO	4	RCTs	Serious^a^	Not serious^d^	Not serious	Serious^c^	Not serious	−0.09 (−0.73, 0.92)	⨁⨁◯◯ low

**Lipid profile**									
TC	4	RCTs	Serious^a^	Not serious	Not serious	Not serious	Not serious	−10.13 (−17.84, −2.41)	⨁⨁⨁◯ moderate
TG	4	RCTs	Serious^a^	Not serious	Not serious	Serious^c^	Not serious	3.42 (−6.39, 13.22)	⨁⨁◯◯ low
HDL-C	3	RCTs	Serious^a^	Not serious	Not serious	Serious^c^	Not serious	1.16 (−1.55, 3.87)	⨁⨁◯◯ low
LDL-C	3	RCTs	Serious^a^	Serious^e^	Not serious	Serious^c^	Not serious	−1.06 (−17.89,15.77)	⨁◯◯◯ very low

**Blood pressure**									
SBP	4	RCTs	Serious^a^	Not serious	Not serious	Not serious	Not serious	3.94 (1.87, 6.01)	⨁⨁⨁◯ moderate
DBP	4	RCTs	Serious^a^	Not serious^f^	Not serious	Serious^c^	Not serious	0.21 (−5.64, 6.05)	⨁⨁◯◯ low

**Glycemic control**									
FBG	4	RCTs	Serious^a^	Not serious	Not serious	Not serious	Not serious	−8.29 (−15.19, −1.39)	⨁⨁⨁◯ moderate

SCr, serum creatinine; BUN, blood urea nitrogen; PRO, proteinuria; TC, total cholesterol; TG, triglycerides; LDL-C, low-density lipoprotein-cholesterol; HDL-C, high-density lipoprotein-cholesterol; SBP, systolic blood pressure; DBP, diastolic blood pressure; FBG, fasting blood glucose; WMD, weight mean difference; CI, confidence interval; RCT, randomized controlled trial. ^a^For risk of bias, the majority of included studies were considered to be at high risk of bias due to the fact that outcomes assessment was not blinded, so it was downgraded. ^b^Although there was substantial heterogeneity for the effect of curcumin on BUN, it was explained when the study conducted by Shafabakhsh et al. was removed according to the sensitivity analysis (original: *I*^2^ = 60%, *P* heterogeneity = 0.082; after study was removed: *I*^2^ = 0%, *P* heterogeneity = 0.601). ^c^The 95% CIs for effect estimates overlap the zero. ^d^Although there was considerable heterogeneity for the effect of curcumin on PRO, it was associated with mean age (≤60 years, *I*^2^ = 41.4%), curcumin intake (≥1500 mg/day, *I*^2^ = 47.4%), and follow-up period (＞2 months, *I*^2^ = 47.4%). ^e^There was substantial heterogeneity (*I*^2^ ≥ 77.2%, *P* < 0.1) that was unexplained by any subgroup or sensitivity analysis for the effect of curcumin on LDL-C. ^f^Although there was substantial heterogeneity, it was explained for the effect of curcumin on DBP with follow-up periods (＞2 months, *I*^2^ = 21.4%).

## Data Availability

The data used to support the findings of this study are included within the article and in the Supplementary Materials.

## Supplementary Materials

Supplementary Materials Table S1: PRISMA 2020 Checklist. Table S2: search strategies of the database. Table S3: Begg's test and Egger's test of curcumin supplementation on outcomes.
